# Nonmetastatic Pulmonary Carcinoid Presenting With Carcinoid Syndrome Despite Negative 5‐HIAA: A Case Report

**DOI:** 10.1155/crie/2260680

**Published:** 2026-02-16

**Authors:** Aditya Chauhan, Muhammed Kizilgul, Emilian Racila, Kidmealem Zekarias

**Affiliations:** ^1^ Department of Medicine, Division of Diabetes, Endocrinology and Metabolism, University of Minnesota, Minneapolis, Minnesota, USA, umn.edu; ^2^ Department of Endocrinology and Metabolism, Etlik City Hospital, University of Health Sciences, Ankara, Türkiye, akdeniz.edu.tr; ^3^ Department of Laboratory Medicine and Pathology, University of Minnesota, Minneapolis, Minnesota, USA, umn.edu

**Keywords:** carcinoid syndrome, case report, pulmonary carcinoid

## Abstract

**Background:**

Carcinoid syndrome from pulmonary carcinoids without hepatic metastases is rare and diagnostically challenging when biochemical markers are normal.

**Case Presentation:**

A woman in her mid‐60 s presented with an incidental right adrenal mass during evaluation for dyspnea and elevated D‐dimer. She reported a 20‐year history of paroxysmal tachycardia and 5 years of episodic flushing, profuse sweating, exertional dyspnea, and fine tremors triggered by minimal physical activity. Imaging revealed a 3.6 cm lipid‐rich adrenal adenoma and an 8 × 10 mm left upper lobe pulmonary nodule. Comprehensive biochemical evaluation was normal: 24 h urinary 5‐hydroxyindoleacetic acid 6 mg/d (normal 0–15), chromogranin A 50 ng/mL (normal 0–187), plasma metanephrine 0.12 nmol/L (normal 0.00–0.49), and normetanephrine 0.68 nmol/L (normal 0.00–0.89), excluding pheochromocytoma. Fine needle aspiration of the left pulmonary nodule confirmed a typical carcinoid tumor. Left upper lobe trisegmentectomy revealed low‐grade neuroendocrine neoplasm (pT1b pN0) with minimal mitotic activity (<1 per 10 HPF) and no necrosis. Postoperative 68Ga‐DOTATATE PET/CT demonstrated a radiotracer‐avid suspicious right lower lobe nodule (SUVmax 2.8) without hepatic metastases. Surgery markedly improved carcinoid syndrome symptoms, with residual episodes responding to long‐acting octreotide 20–30 mg every 4 weeks.

**Discussion:**

This case illustrates that pulmonary carcinoid tumors can present with carcinoid syndrome despite normal biochemical markers and the absence of hepatic metastases. The temporal improvement following resection and response to octreotide established the diagnosis when biochemical testing was uninformative.

**Conclusion:**

Clinicians should maintain high suspicion for pulmonary carcinoids in patients with unexplained paroxysmal symptoms, even with negative biochemical testing and absent metastatic disease.

## 1. Introduction

Carcinoid tumors are rare neuroendocrine neoplasms derived from enterochromaffin cells, predominantly arising in the gastrointestinal tract [1, 2]. Pulmonary carcinoids account for 20%–30% of all carcinoid tumors but represent only 1%–2% of all lung neoplasms [2, 3]. These tumors typically present with symptoms related to bronchial obstruction, including obstructive pneumonitis, pleuritic pain, atelectasis, and dyspnea [4].

Carcinoid syndrome—characterized by flushing, diaphoresis, tachycardia, and dyspnea—occurs in ~8%–30% of patients with carcinoid tumors overall [5– 7]. However, carcinoid syndrome is rare in pulmonary carcinoids and typically occurs only in the presence of hepatic metastases. This rarity is illustrated by a large series of 142 pulmonary carcinoid cases in which only one patient developed carcinoid syndrome, and that patient had documented liver metastasis [8].

We report a case of pulmonary carcinoid presenting with carcinoid syndrome in the absence of hepatic metastases and with negative biochemical markers. The patient experienced significant symptomatic improvement following surgical resection.

## 2. Case Presentation

This case report follows the CARE guidelines (Supporting Information). A woman in her mid‐60s was referred to our endocrinology clinic following the incidental discovery of an adrenal mass on chest computed tomography performed for evaluation of shortness of breath and elevated D‐dimer.

The patient reported a 5‐year history of episodic symptoms, including profuse sweating, exertional dyspnea, occasional dizziness, and fine bilateral hand tremors, often triggered by minimal physical activity. She also described a 20‐year history of paroxysmal tachycardia with heart rates reaching 120–170 beats per minute, previously managed with metoprolol and attributed to anxiety and panic attacks. Additionally, she experienced intermittent facial and neck flushing characterized by visible erythema lasting several minutes to 1 h, frequently accompanied by diaphoresis, tachycardia, and exertional dyspnea.

Past medical history included prediabetes, hyperlipidemia, laryngopharyngeal reflux disease, right ureteral stone, anxiety, migraines, and tinnitus. Family and social history were unremarkable.

Physical examination revealed stable vital signs (BP: 122/79 mmHg, HR: 81 bpm) and a BMI of 32.8 kg/m^2^. Cardiovascular examination demonstrated regular rate and rhythm. Pulmonary, abdominal, neurological, and mood assessments were unremarkable. Current medications included metoprolol succinate ER 125 mg daily and duloxetine 60 mg daily. The patient had no known drug allergies.

### 2.1. Investigations

#### 2.1.1. Imaging Studies

Initial chest CT identified an 8 × 10 mm hypodense left upper lobe perihilar nodule (Figure 1A) and an incidental 3.5 cm right adrenal mass. Dedicated adrenal protocol imaging confirmed a 3.6 cm homogeneous right adrenal mass with attenuation values <10 Hounsfield units, consistent with a lipid‐rich lesion.

Figure 1(A) Preoperative chest CT showing an 8 × 10 mm hypodense left upper lobe perihilar nodule (red arrow). (B) Preoperative FDG‐PET/CT demonstrating a 1.1 cm left upper lobe perihilar nodule with FDG uptake similar to blood pool (SUVmax 2.8) (white arrow). (C) Postoperative DOTATATE PET/CT revealing an 8 mm smoothly marginated pulmonary nodule in the right lower lobe adjacent to a vessel, demonstrating mild radiotracer uptake with SUVmax 2.9 (white arrow).

**Figure:**
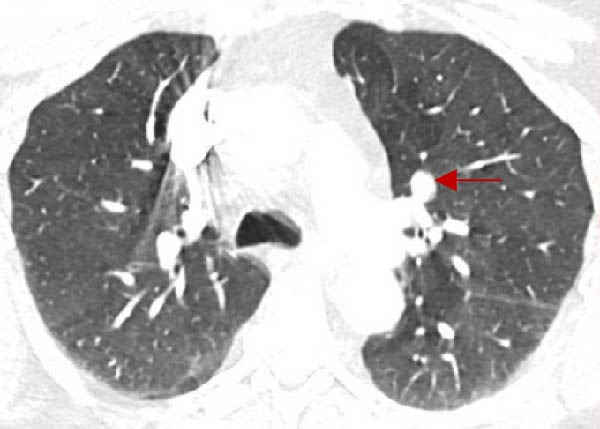
(A)

**Figure:**
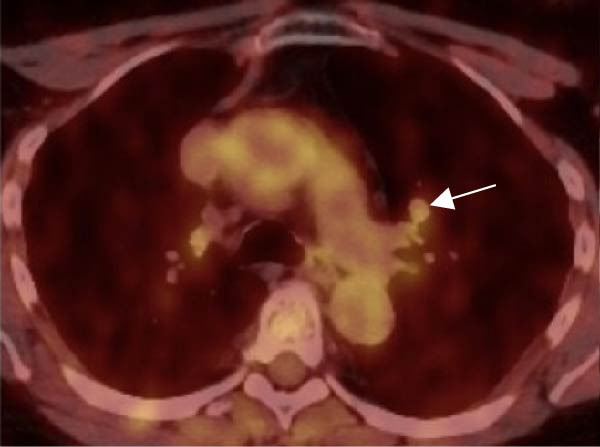
(B)

**Figure:**
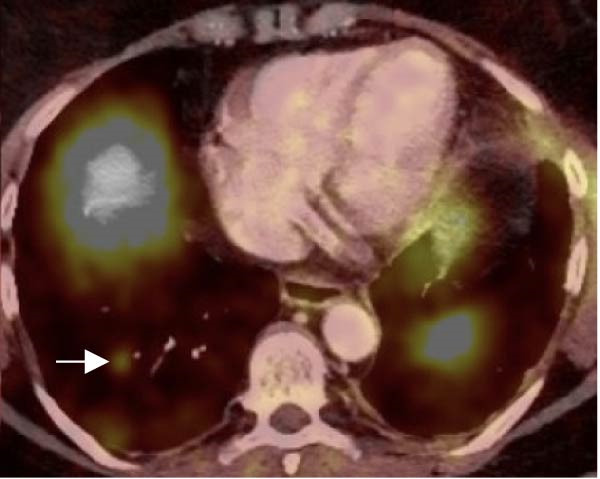
(C)

18 F‐FDG‐PET/CT demonstrated minimal metabolic activity in the right adrenal mass (SUVmax 2.9, below hepatic background) and a 1.1 cm left upper lobe perihilar nodule with FDG uptake similar to blood pool (SUVmax 2.8) (Figure 1B). Follow‐up chest CT 4 months later showed persistence of the 1.1 cm left upper lobe nodule and a new 3 mm solid nodule in the right lower lobe.

#### 2.1.2. Biochemical Evaluation

Neuroendocrine markers were assessed given clinical suspicion for carcinoid syndrome. Twenty‐4‐h urinary 5‐hydroxyindoleacetic acid (5‐HIAA) levels measured 6 mg/d (reference 0–15 mg/d; 31.4 μmol/d, 0–78.5 μmol/d) with a 5‐HIAA/creatinine ratio of 4 mg/gCR (reference 0–14 mg/gCR; 2.1 μmol/mmol, 0–7.4 μmol/mmol), findings consistent across two measurements. Chromogranin A was normal at 50 ng/mL (reference 0–187 ng/mL).

Given the presentation of tachycardia, diaphoresis, and adrenal mass, catecholamine‐secreting tumors were investigated. Plasma metanephrines showed metanephrine 0.12 nmol/L (reference 0.00–0.49 nmol/L) and normetanephrine 0.68 nmol/L (reference 0.00–0.89 nmol/L). Twenty‐4‐h urine metanephrines were within normal limits, effectively excluding a clinically significant catecholamine‐producing tumor.

Additional adrenal functional testing, including the dexamethasone suppression test, aldosterone, 17‐hydroxyprogesterone, testosterone, 11‐deoxycortisol, and DHEA‐S, was unremarkable.

#### 2.1.3. Tissue Diagnosis

Fine needle aspiration of the left lung nodule confirmed a neuroendocrine (carcinoid) tumor.

### 2.2. Differential Diagnosis

The combination of paroxysmal symptoms and adrenal mass initially suggested pheochromocytoma. However, comprehensive biochemical evaluation with normal plasma and urine metanephrines excluded catecholamine excess. The lipid‐rich adrenal mass (attenuation <10 HU) and low FDG uptake (SUVmax 2.9) supported a benign adenoma.

Carcinoid syndrome appeared unlikely given normal biomarkers. Both 5‐HIAA (6 mg/d) and chromogranin A (50 ng/mL) were within reference ranges despite classic clinical symptoms, creating diagnostic uncertainty.

Psychiatric or medication‐induced etiology was considered given the patient’s anxiety history and duloxetine therapy, as this medication is known to cause flushing. However, her symptoms demonstrated an unpredictable episodic pattern lasting up to 1 h and triggered by minimal activity, rather than the consistent, dose‐related timing (1–2 h post‐administration) typical of medication effects. Hyperthyroidism was excluded through normal thyroid function studies.

### 2.3. Treatment

The patient underwent a left upper lobe trisegmentectomy, which revealed a typical carcinoid tumor (pT1b, pN0) consistent with a low‐grade neuroendocrine neoplasm. Histopathology demonstrated minimal mitotic activity (<1 per 10 high‐power fields) and no necrosis. All 14 resected lymph nodes were negative for malignancy (Figure 2).

Figure 2Histopathology of lung left upper lobe carcinoid tumor. Carcinoid tumors may develop bronchiolocentrically or peripherally. At low magnification (20×, A), the tumor (solid arrow) is well circumscribed, partly encapsulated, and located next to a large bronchial branch in this patient (open arrow). Carcinoid tumors usually have an organized architecture, with neoplastic cells either forming large nests or showing a trabecular growth pattern. No tumor necrosis is observed (100×, B). At high magnification, tumor cells have a granular, “salt and pepper” chromatin, with very small or inconspicuous nucleoli. Only rare mitoses are present in typical carcinoid tumors, as in this case (0–1 mitoses per 10 high‐power fields). Scattered small‐sized lymphocytes characterized by nuclei with condensed chromatin and a small amount of cytoplasm are observed in between neoplastic cells (400×, C). In this typical carcinoid tumor, the spread of tumor clusters (solid arrows) through airway spaces (STAS) lined by respiratory pseudocolumnar ciliated epithelium (open arrow) was identified. (100×, D). The tumor is characteristically diffusely and strongly positive for neuroendocrine markers (Chromogranin IHC, 100×, E). Typical carcinoid tumors have a low overall proliferation index, generally less than 5% overall (Ki‐67 IHC, 100×, F).

**Figure:**
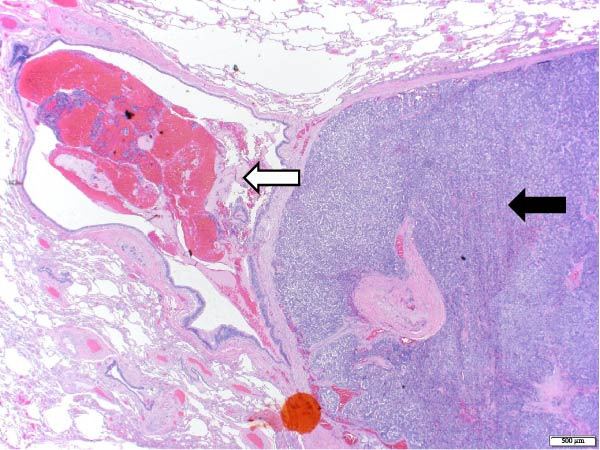
(A)

**Figure:**
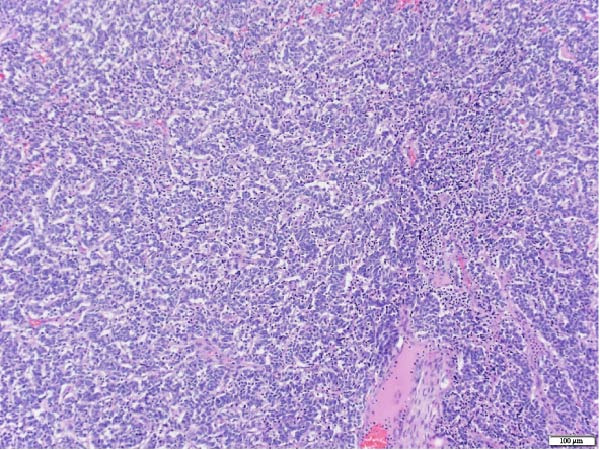
(B)

**Figure:**
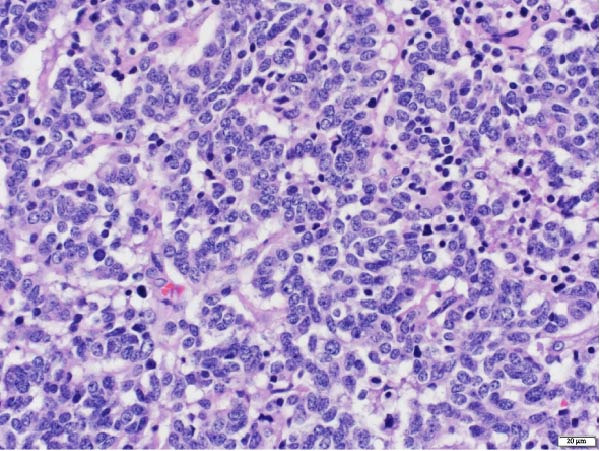
(C)

**Figure:**
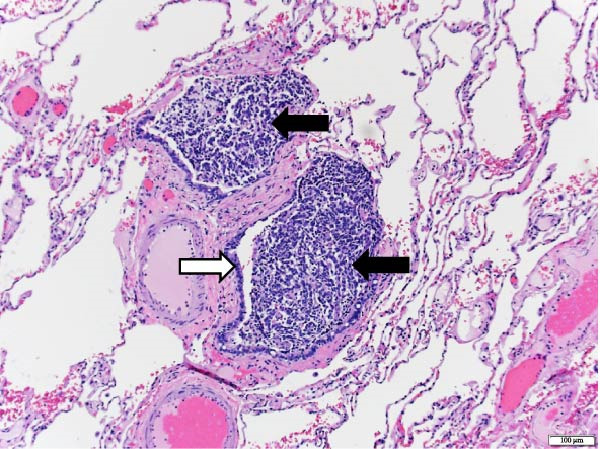
(D)

**Figure:**
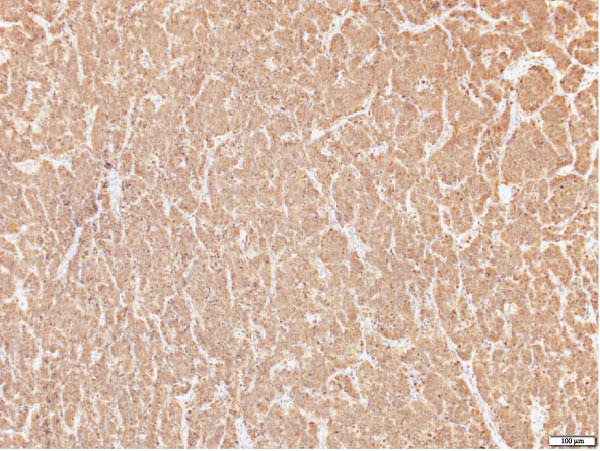
(E)

**Figure:**
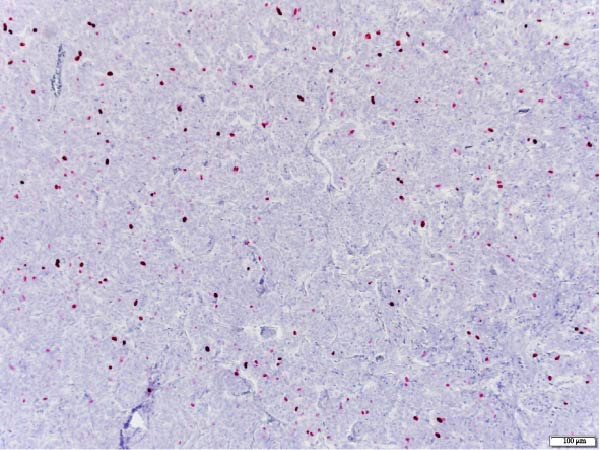
(F)

Postoperative 68Ga‐DOTATATE PET/CT demonstrated mild somatostatin receptor uptake (SUVmax 2.8) in an 8 mm right lower lobe nodule, suspicious for a second carcinoid tumor (Figure 1C). No abnormal uptake was observed elsewhere, including the liver, suggesting the absence of distant metastases. Follow‐up imaging at six and 12 months demonstrated stability of this nodule without new sites of uptake.

### 2.4. Outcome and Follow‐up

Following surgery, the patient reported marked improvement in carcinoid syndrome symptoms, including episodic flushing, diaphoresis, tachycardia, and exertional dyspnea. She continued to experience occasional flushing and exertional diaphoresis. Sandostatin LAR 20 mg every 4 weeks was initiated, resulting in further improvement with symptoms reduced to two to three episodes per week after two injections. Given this favorable response and good tolerability, the dose was increased to 30 mg every 4 weeks.

The temporal improvement following initial resection, combined with response to Sandostatin LAR and unremarkable alternative diagnostic evaluations, supported the diagnosis of carcinoid syndrome originating from a pulmonary neuroendocrine tumor.

Long‐term follow‐up imaging at 1‐year post‐surgery demonstrated continued stability of the right lower lobe nodule. Repeat 68Ga‐DOTATATE PET/CT showed persistent but stable subtle radiotracer activity in this nodule, consistent with a likely second carcinoid tumor. The patient continues on surveillance imaging given the indeterminate but stable nature of this lesion. The previously identified right adrenal mass remained stable at 3.2 cm without radiotracer uptake, confirming its benign nature. No new sites of disease were identified, and the patient remained clinically stable on octreotide therapy.

## 3. Discussion

This case illustrates the diagnostic challenges of a pulmonary carcinoid tumor presenting with classic carcinoid syndrome in the absence of hepatic metastases and with negative biochemical markers.

### 3.1. Epidemiological Context

Pulmonary carcinoids demonstrate distinct clinical behavior compared with their gastrointestinal counterparts. In a five‐decade analysis of 13,715 carcinoid tumors, bronchopulmonary carcinoids represented 25.3% of all cases and typically presented with local respiratory symptoms including cough, wheezing, hemoptysis, dyspnea, recurrent pneumonia, and chest pain [2, 7].

Carcinoid syndrome occurs uncommonly in lung carcinoids and is usually limited to cases with hepatic metastases [7, 8]. In a comprehensive Israeli series of 142 pulmonary carcinoid cases, only one patient developed carcinoid syndrome, and that patient had documented liver metastasis [8]. Population‐based studies support this observation, reporting carcinoid syndrome in ~8% of lung carcinoid patients—substantially lower than rates observed in small bowel NETs (32%) or across all NET types (19%) [9].

### 3.2. Pathophysiological Considerations

The rarity of carcinoid syndrome in pulmonary NETs reflects two key factors: the high concentration of monoamine oxidase in the pulmonary system, which metabolizes serotonin locally, and the relatively infrequent occurrence of distant metastases in these patients [2, 5–8, 10]. Although bioactive substances secreted by neuroendocrine tumors, particularly serotonin, drive carcinoid syndrome symptoms [9, 11], the diagnostic sensitivity of biochemical markers remains limited.

The pathophysiology underlying carcinoid syndrome with normal biochemical markers remains incompletely understood. Interpretation of 24 h urinary 5‐hydroxyindoleacetic acid (5‐HIAA) requires careful consideration of multiple interference factors [12, 13]. False‐negative results may occur in patients with clinically evident carcinoid syndrome, particularly when diarrhea is absent or when tumors predominantly secrete vasoactive substances other than serotonin. Medications can interfere with 5‐HIAA measurement: some agents (including corticosteroids, methyldopa, and certain psychotropic medications) may lower levels, while others (such as acetaminophen, caffeine, and reserpine) can cause false‐positive elevations [13].

Dietary sources rich in serotonin or its precursors—including avocados, bananas, walnuts, tomatoes, and certain fruits—can significantly elevate urinary 5‐HIAA if consumed during the collection period, necessitating dietary restriction for at least 72 h before and during collection [13]. Additionally, impaired renal function may result in falsely low urinary excretion despite elevated systemic serotonin levels, and technical factors such as incomplete collection or improper specimen handling can affect assay accuracy [12, 13].

In the present case, renal function was preserved, dietary and medication interferences were excluded, and urine collection was appropriately performed, supporting the conclusion that normal 5‐HIAA levels did not exclude active carcinoid syndrome.

### 3.3. Therapeutic Strategies and Precision Medicine Considerations

Management of NETs is guided by tumor differentiation, disease extent, functional status, and somatostatin receptor expression. Surgical resection remains the only curative option for localized pulmonary carcinoid tumors and may provide symptomatic relief in patients with functional disease. Somatostatin analogs (SSAs), including octreotide and lanreotide, serve as first‐line therapy for hormone‐related symptoms and have demonstrated antiproliferative benefits in patients with well‐differentiated NETs [7].

Additional treatment modalities include peptide receptor radionuclide therapy (PRRT) for somatostatin receptor–positive tumors [14, 15], targeted therapies for progressive disease, and chemotherapy for high‐grade neoplasms. Precision medicine approaches incorporating molecular profiling are increasingly guiding personalized treatment strategies in NET management [16].

### 3.4. Clinical Implications

This case provides several important clinical lessons. First, pulmonary carcinoid tumors can manifest with carcinoid syndrome even in the absence of hepatic metastases. Second, biochemical testing can be negative in symptomatic carcinoid syndrome, and the diagnosis should not be excluded based solely on normal biomarker results.

Carcinoid tumors frequently present with nonspecific symptoms that lead to significant diagnostic delays. Clinicians should maintain a high index of suspicion for these neoplasms in patients with unexplained flushing, even when typical biomarkers are normal [17, 18]. While carcinoid syndrome with normal 5‐HIAA has been reported previously, this case contributes to the limited literature by demonstrating the diagnostic challenges when biomarkers are negative, liver metastases are absent, and multiple potential etiologies coexist in patients with paroxysmal symptoms.

## Author Contributions

**Aditya Chauhan**: manuscript writing, literature review. **Muhammed Kizilgul:** manuscript writing, literature review and editing. **Emilian Racila:** pathological analysis, provision of histopathology images and interpretation. **Kidmealem Zekarias:** supervision of patient care, manuscript writing and editing, critical revision for important intellectual content, final approval of the manuscript.

## Acknowledgments

During the preparation of this work, the authors used claude.ai in order to improve readability and language.

## Funding

No funding was received for this work.

## Disclosure

All authors have read and approved the final version of the manuscript. Dr. Kidmealem Zekarias had full access to the case report and takes complete responsibility for the integrity of the data and the accuracy of the data. After using claude.ai tool, the authors reviewed and edited the content as needed and take full responsibility for the content of the publication.

## Ethics Statement

This case report was conducted in accordance with institutional ethical guidelines. Formal ethics committee approval was not required for this single case report per institutional policy.

## Consent

Written informed consent was obtained from the patient for publication of this case report and any accompanying images.

## Conflicts of Interest

The authors declare no conflicts of interest.

## Supporting Information

Additional supporting information can be found online in the Supporting Information section.

## Supporting information


**Supporting Information** Completed CARE checklist documenting adherence to CARE guidelines for case report methodology and transparent reporting.

## Data Availability

Data sharing is not applicable to this article, as no datasets were generated or analyzed during the current study.
