# Supplementary material for: Nonmetastatic Pulmonary Carcinoid Presenting With Carcinoid Syndrome Despite Negative 5‐HIAA: A Case Report
Source: Case Rep Endocrinol. 2026 Feb 16;2026:2260680. doi: 10.1155/crie/2260680 (PMC12907734; doi:10.1155/crie/2260680)
Supplement: Supplementary file 1 — Supporting Information Completed CARE checklist documenting adherence to CARE guidelines for case report methodology and transparent reporting. [file CRIE-2026-2260680-s001.docx]

**CARE Checklist**

CAse REport (CARE) Guidelines Checklist of Information to Include When Writing a Case Report

Manuscript Title: Non-Metastatic Pulmonary Carcinoid Presenting with Carcinoid Syndrome Despite Negative 5-HIAA

| **Section/Topic** | **Item** | **Checklist Item Description** | **Page #** | **Present** |
| --- | --- | --- | --- | --- |
| Title | 1 | The words 'case report' (or 'case study') should be in the title | 1 | Yes |
| Keywords | 2 | 2 to 5 key words that identify topics in this case report | 1 | Yes |
| Abstract | 3a | Introduction - What is unique and why is it important? | 1 | Yes |
|  | 3b | Patient's main concerns and important clinical findings | 1 | Yes |
|  | 3c | The main diagnoses, interventions, and outcomes | 1 | Yes |
|  | 3d | The main 'take-away' lessons from this case report | 1 | Yes |
| Introduction | 4 | Brief background summary of the case with references | 2 | Yes |
| Patient Information | 5a | De-identified demographic and other patient-specific information | 2-3 | Yes |
|  | 5b | Main symptoms, including duration | 2-3 | Yes |
|  | 5c | Medical, family, and psychosocial history | 3 | Yes |
| Clinical Findings | 6 | Describe the relevant physical examination (PE) findings | 3 | Yes |
| Timeline | 7 | Important dates and times (as table, figure, or narrative) | 2-3 | Yes |
| Diagnostic Assessment | 8a | Diagnostic methods (e.g., PE, laboratory, imaging, surveys) | 3-4 | Yes |
|  | 8b | Diagnostic challenges (e.g., access, financial, cultural) | 4 | Yes |
|  | 8c | Diagnostic reasoning including other diagnoses considered | 4 | Yes |
|  | 8d | Prognostic characteristics (such as staging) | 4-5 | Yes |
| Therapeutic Intervention | 9a | Types of intervention (e.g., pharmacologic, surgical, preventive) | 4-5 | Yes |
|  | 9b | Administration of intervention | 5 | Yes |
|  | 9c | Changes in intervention with explanations | 5 | Yes |
| Follow-up and Outcomes | 10a | Clinician and patient-assessed outcomes (when appropriate) | 5 | Yes |
|  | 10b | Important follow-up test results (positive or negative) | 5 | Yes |
|  | 10c | Intervention adherence and tolerability | 5 | Yes |
|  | 10d | Adverse and unanticipated events | 5 | Yes |
| Discussion | 11a | Discussion of the strengths and limitations in management | 5-6 | Yes |
|  | 11b | Discussion of relevant medical literature | 5-6 | Yes |
|  | 11c | Rationale for conclusions (including assessment of cause and effect) | 6 | Yes |
|  | 11d | Primary 'take-away' lessons from this case report | 6 | Yes |
| Patient Perspective | 12 | Patient should share their perspective or experience whenever possible | N/A | No |
| Informed Consent | 13 | Did the patient give informed consent? Please provide if requested | 7 | Yes |

*Note: Item 12 (Patient Perspective) is not included as the patient's direct perspective was not formally solicited for this clinical case report. All other CARE guideline items have been addressed.*

Reference: Riley DS, Barber MS, Kienle GS, et al. CARE guidelines for case reports: explanation and elaboration document. J Clin Epidemiol. 2017;89:218-235.

Available at: https://www.care-statement.org
